# Lineage-dependent differences in the disease progression of Zika virus infection in type-I interferon receptor knockout (A129) mice

**DOI:** 10.1371/journal.pntd.0005704

**Published:** 2017-07-03

**Authors:** Stuart D. Dowall, Victoria A. Graham, Emma Rayner, Laura Hunter, Barry Atkinson, Geoff Pearson, Mike Dennis, Roger Hewson

**Affiliations:** National Infection Service, Public Health England, Porton Down, Salisbury, Wiltshire, United Kingdom; Baylor College of Medicine, UNITED STATES

## Abstract

Zika virus (ZIKV) falls into two lineages: African (ZIKV^AF^) and Asian (ZIKV^AS^). These lineages have not been tested comprehensively in parallel for disease progression using an animal model system. Here, using the established type-I interferon receptor knockout (A129) mouse model, it is first demonstrated that ZIKV^AF^ causes lethal infection, with different kinetics of disease manifestations according to the challenge dose. Animals challenged with a low dose of 10 plaque-forming units (pfu) developed more neurological symptoms than those challenged with 5-log higher doses. By contrast, animals challenged with ZIKV^AS^ displayed no clinical signs or mortality, even at doses of 10^6^ pfu. However, viral RNA was detected in the tissues of animals infected with ZIKV strains from both lineages and similar histological changes were observed. The present study highlights strain specific virulence differences between the African and Asian lineages in a ZIKV mouse model.

## Author summary

Since first being recognised in 1947, Zika virus (ZIKV) has mainly been associated with a mild illness with symptoms including a limited fever and rash. In 2007 the virus spread from Africa and Asia into Micronesia, then in 2013 into French Polynesia and then onwards across Pacific regions and into South America. In these new regions, ZIKV has been associated with more severe clinical conditions including Gullain-Barre syndrome (GBS) and congenital Zika syndrome. Using a mouse strain with a deficiency in the type-I interferon receptor (A129), after challenge with ZIKV using a route that resembles the natural route of infection via mosquito bite we compared the two major lineages of ZIKV: African (ZIKA^AF^) and Asian (ZIKV^AS^). Whilst it was known that ZIKV^AF^ causes a lethal disease in A129 mice, we observed a non-lethal infection with ZIKV^AS^. To confirm the finding, a recent isolate of ZIKV^AS^ was additionally assessed and demonstrated the same observations. Our studies provide new insights into the mechanisms of ZIKV infection in a small animal model; and may help to elucidate the different pathologies caused by this virus.

## Introduction

Zika virus (ZIKV) is a flavivirus which was first isolated from a sentinel rhesus macaque placed in the Zika forest in Uganda in 1947 [1] and later from African mosquitoes collected in the same forest in the early 1960’s [2]. The virus remained a local curiosity of the East African Virus Research Institute, Entebbe, being noted for its febrile, but mild and unproblematic self-limiting symptoms in humans [3], for several years. Subsequent studies went on to show evidence of its wide circulation, notably without serious symptoms, in several African and Asian countries during the 1960s to 1980s [4–7]. However, in 2007 an outbreak on Yap Island, Micronesia, in the Pacific ocean, changed the ZIKV landscape with the first reports of infection outside Africa and Asia [8]. No further transmission was identified until 2013 when French Polynesia reported autochthonous cases [9] and a large outbreak [10]. The virus continued to spread rapidly throughout the Pacific region [11] before being detected in Brazil, from where it spread to other countries across South America [12, 13]. With this spread into new territories came newly identified pathological changes attributed to ZIKV infection, including microcephaly [14, 15] (now recently recognised as congenital Zika syndrome) and Guillain-Barre syndrome [16]. This increase in disease severity caused the World Health Organisation to declare ZIKV a Public Health Emergency of International Concern (PHEIC) in February, 2016 [17, 18] which was subsequently removed in November 2016.

While several reports demonstrate sexual transmission of ZIKV [19] and blood/platelet transfusion [20], the main route of infection is via mosquito bites. Ideally, *in vivo* models should be developed which closely mirror natural infection. Subcutaneous inoculation is a common method used for studying mosquito-transmitted pathogens as it mimics a natural route of infection, including local replication at the inoculation site. Whilst the tropism of ZIKV is not yet fully understood, it is likely that keratinocytes and dendritic cells in the skin represent early targets of infection [21], as occurs for other flaviviruses such as Dengue 1–4 viruses [22, 23] and West Nile virus [24].

Although NHP models for ZIKV are available, small animal models are valuable for the initial assessment of safety, immunogenicity, and protective efficacy of candidate vaccines prior to testing in NHPs and subsequent human clinical trials [25]. Small animal models for ZIKV infection have focused on mice with deficiencies in their IFN response, since the virus has been demonstrated to target human STAT2 proteins to suppress IFN signalling, but not mouse STAT2 [26]. Lethal models have been developed using mice with deficiencies in their type-I interferon receptor on a 129Sv/Ev background (A129) [27, 28] and with other parental background strains (*Ifnar1*^*-/-*^) [29–31]. To develop a wild-type (WT) mouse model of ZIKV infection, antibody treatment to block type-I IFN signalling has been used to replicate the phenotype of the A129/*Ifnar1*^*-/-*^ mice. After challenge with an Asian strain (H/PF/2013) of ZIKV, higher viral loads were observed in WT mice pre-treated with the antibody, but there was no lethality or loss in weight [30]. This mouse model has also been challenged with a mouse-adapted African strain (Dakar) of ZIKV with virus induced lethality being observed from days 10 to 15 post-challenge in some, but not all, of the control treated animals. This model was also used to assess the efficacy of monoclonal antibody therapy after subcutaneous challenge with 10^3^ FFU ZIKV (Dakar) [32]. In a different study to assess ZIKV-induced damage to the testis, however, the same model infected with a 3 log higher dose of ZIKV (Dakar) reported no lethality [29]. Thus, while the WT mouse model has been useful it also appears to give inconsistent results with certain strains of ZIKV. Additionally, while virus adaptation to the mouse by serial passage of ZIKV was used in 1952 to develop the original murine model [33], the approach has the potential to alter virulence and antigenicity of the virus, therefore compromising any model developed from it [25]. Since animal models need to be consistent and reproducible between laboratories, with the minimum of changes needed to replicate natural disease, the A129 mouse in conjunction with natural strains remains a valuable model for the study of ZIKV infection.

ZIKV is phylogenetically divided into two lineages: African and Asian [34, 35]. Differences in pathogenicity between ZIKV’s of the Africa (ZIKV^AF^) and Asia (ZIKV^AS^) lineages have not been reported in A129 mice. To this end, we have conducted a series of experiments to investigate the different disease outcomes and pathological changes in A129 mice challenged with ZIKV^AF^ and ZIKV^AS^ via the subcutaneous route, to mimic mosquito-bite infection.

## Results

### ZIKV causes dose-dependent disease kinetics in A129 mice

Whilst it has been demonstrated that A129 mice are susceptible to a 10^6^ plaque-forming unit (pfu) subcutaneous dose of ZIKV^AF^ infection [27], their susceptibility to lower challenge doses by this route is not known. A dose reduction study was conducted with challenge doses ranging from 10^6^–10 pfu. Virus challenge was delivered subcutaneously in order to mimic natural infection via mosquito bite [36], and included the range of 10^4^–10^6^ pfu which has been implicated for infection with West Nile virus, another mosquito-borne flavivirus [37].

**Fig 1:**
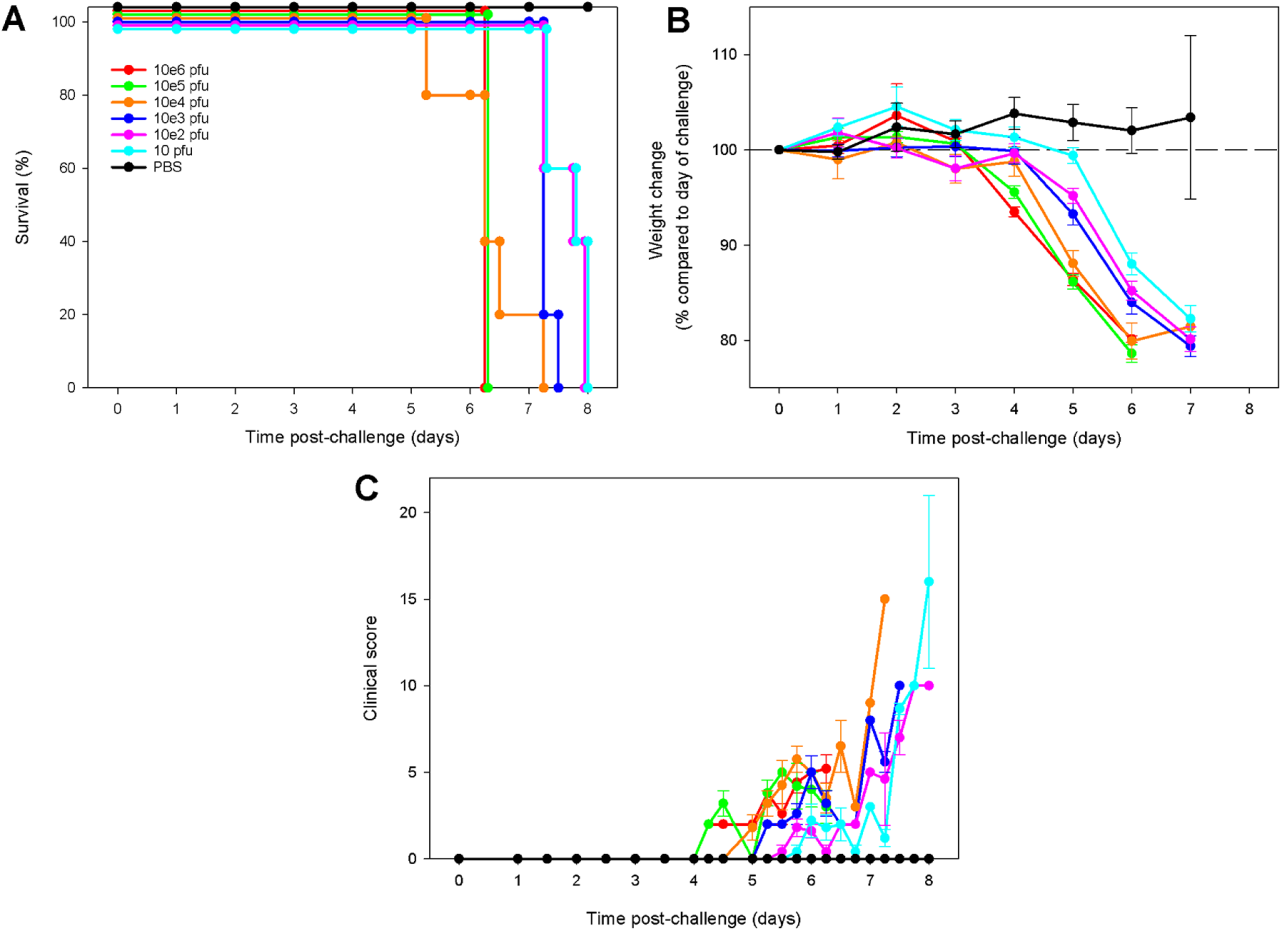
Clinical data from A129 mice challenged with different doses of ZIKV^AF^. 6–8 week old A129 mice were subcutaneously challenged with 10^6^, 10^5^, 10^4^, 10^3^, 10^2^ or 10 pfu ZIKV^AF^ virus. (A) Kaplin-Meier survival plot. (B) Differences in weight compared to date of challenge. (C) Clinical score, with numerical values given as follows: 0, normal; 2, ruffled fur; 3, lethargy, pinched, hunched, wasp waisted; 5, laboured breathing, rapid breathing, inactive, neurological; and 10, immobile. Graphs B and C show the mean values with error bars denoting standard error. Group sizes were n = 5.

All ZIKV^AF^-challenged mice lost weight, succumbed to infection and met humane clinical endpoints within 8 days (Fig 1). Clinical signs in the mice were recorded at least twice a day and given a numerical value according to severity. Both weight loss and lethality were dose dependent, with animals receiving the lower doses surviving longer and losing weight at later time points. Mice challenged with higher doses of ZIKV^AF^ survived for less time and developed fewer clinical signs than those receiving lower concentrations (Fig 1C). As a result of the increased length of the disease progression in mice challenged with 10 pfu ZIKV^AF^, clinical disease in these animals appeared more severe with neurological signs observed in several animals.

### ZIKV^AF^ is pathogenic to A129 mice; ZIKA^AS^ does not cause signs of illness, although virus is detectable

**Fig 2:**
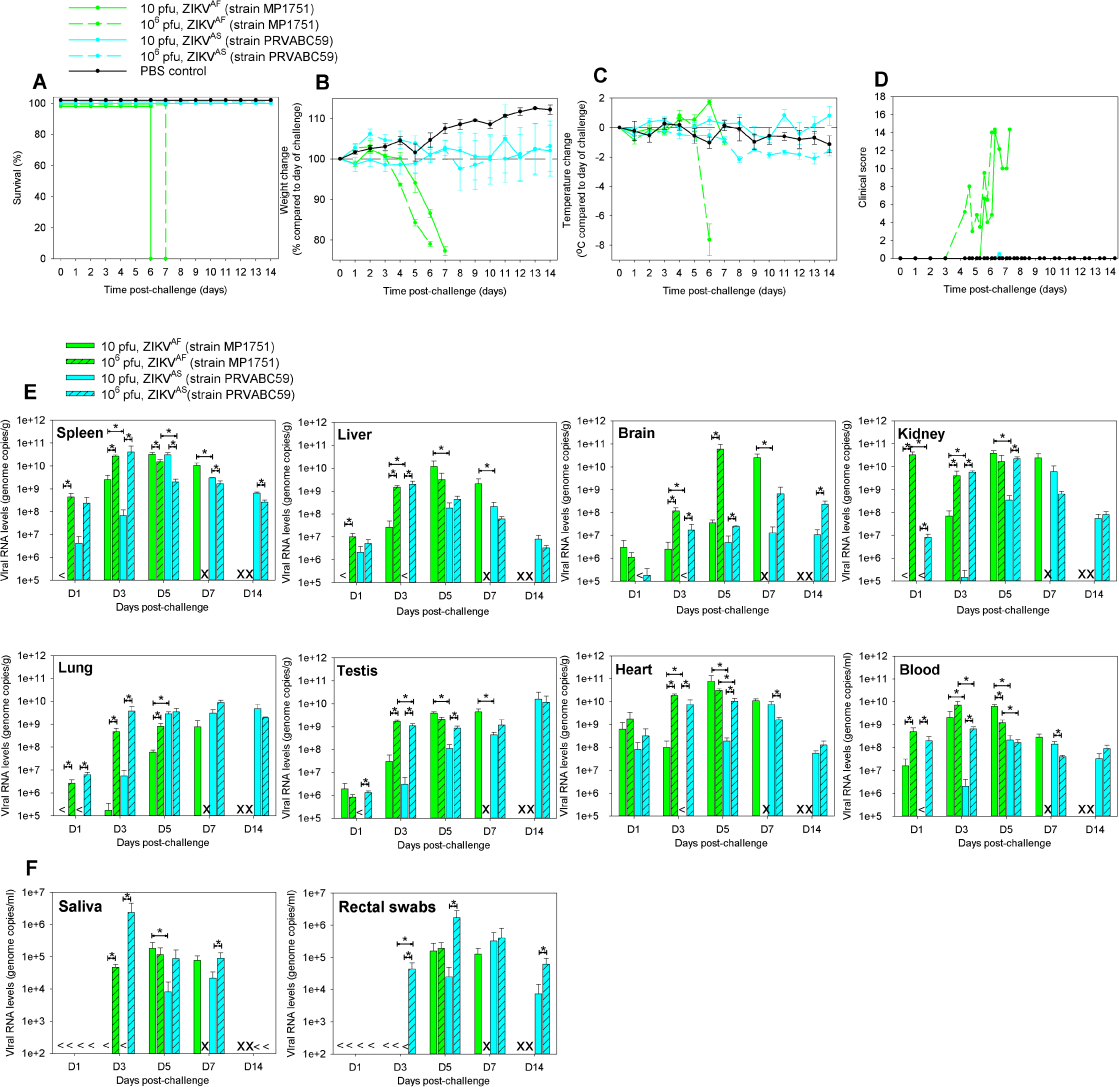
Clinical data and viral burden from A129 mice challenged with ZIKV^AF^ and ZIKV^AS^. 6–8 week old A129 mice were subcutaneously challenged with a high (10^6^ pfu) or low (10 pfu) dose of ZIKV^AF^ or ZIKV^AS^. At days 1, 3, 5 and 7 post-challenge, a cohort of mice from each group were culled for assessment of local response. (A) Kaplin-Meier survival plot. (B) Differences in weight compared to day of challenge. (C) Differences in temperatures compared to day of challenge. (D) Clinical score, with numerical values given as follows: 0, normal; 2, ruffled fur; 3, lethargy, pinched, hunched, wasp waisted; 5, laboured breathing, rapid breathing, inactive, neurological; and 10, immobile. (E) Viral burden in local tissues (spleen, liver, brain, kidney, lung, testes, heart and blood) at days 1, 3, 5, 7 and 14 post-challenge. (F) Viral burden in secretions (saliva and rectal swabs) of animals at days 1, 3, 5, 7 and 14 post-challenge. Graphs A-D: group sizes were n = 6.Graphs B—D show the mean values with error bars denoting standard error. Graphs E-F: groups sizes of n = 3, with bar denoting mean values and error bars denoting standard error. Abbreviations: <, below the limit of detection; x, no results as animals had previously met humane endpoints; and *, statistical significance (P = 0.0809, Mann-Whitney test).

To ascertain the differences between the two lineages of ZIKV, A129 mice were challenged with high and low doses (10^6^ and 10 pfu, respectively) of each strain. All animals challenged with ZIKV^AF^ met humane endpoints, whereas those challenged with ZIKV^AS^ survived the 14 days of the study (Fig 2A). Weight loss in the ZIKV^AF^-challenged group was observed, whereas those which received ZIKV^AS^ neither lost nor gained weight compared to unchallenged controls (Fig 2B). Animals which received the highest dose of ZIKV^AF^ demonstrated profound decreases in temperature prior to meeting humane endpoints (Fig 2C). Similarly, only those animals challenged with ZIKV^AF^ had substantial clinical signs (Fig 2D).

To follow up the clinical observations at days 1, 3, 5, 7 and 14 post-challenge, a cohort of mice were culled and viral RNA levels were determined at local sites (Fig 2E). In the spleen and liver, similar viral RNA levels were seen between the dose-matched groups. In the brain, both ZIKV^AF^-challenged groups showed viral loads detectable from day 1, yet for the low dose ZIKV^AS^ animals, viral RNA was only detectable at day 5. The viral RNA levels in the brains of ZIKV^AF^-challenged groups were consistently higher than those in the brains of ZIKV^AS^-challenged groups. Evidence of viral RNA in the kidney and lung were observed with both lineages, although in both tissues, animals challenged with only the low dose having detectable concentrations 3 days post-challenge. In the testis, similar levels were observed between the two strains. The levels in the ZIKV^AS^-challenged group increased continually over the 14 day study period. In the heart and blood, similar kinetics were observed between the ZIKV strains with the levels peaking on days 3 and 5, respectively, and then decreasing at later time points. These results demonstrate that both strains of ZIKV caused infection in the mice with evidence of systemic virus spread, most likely haematogenously.

To monitor for virus shedding, saliva and rectal swabs were collected and viral RNA levels were assessed (Fig 2F). Viral RNA was detectable in the saliva in all groups at day 5, but at earlier time points only in animals challenged with the high dose inoculum. Observations with the rectal swabs were similar, although viral RNA was only observed on day 3 in the high dose ZIKV^AS^ group. Viral RNA did not appear in the other groups until day 5. Whilst the level of viral RNA in the secreted components was lower than those detected at the local sites, the data provide evidence that ZIKV is present in secretions.

### Histological changes in the brain were observed at earlier time points after ZIKV^AF^ infection then after ZIKV^AS^ infection

**Fig 3:**
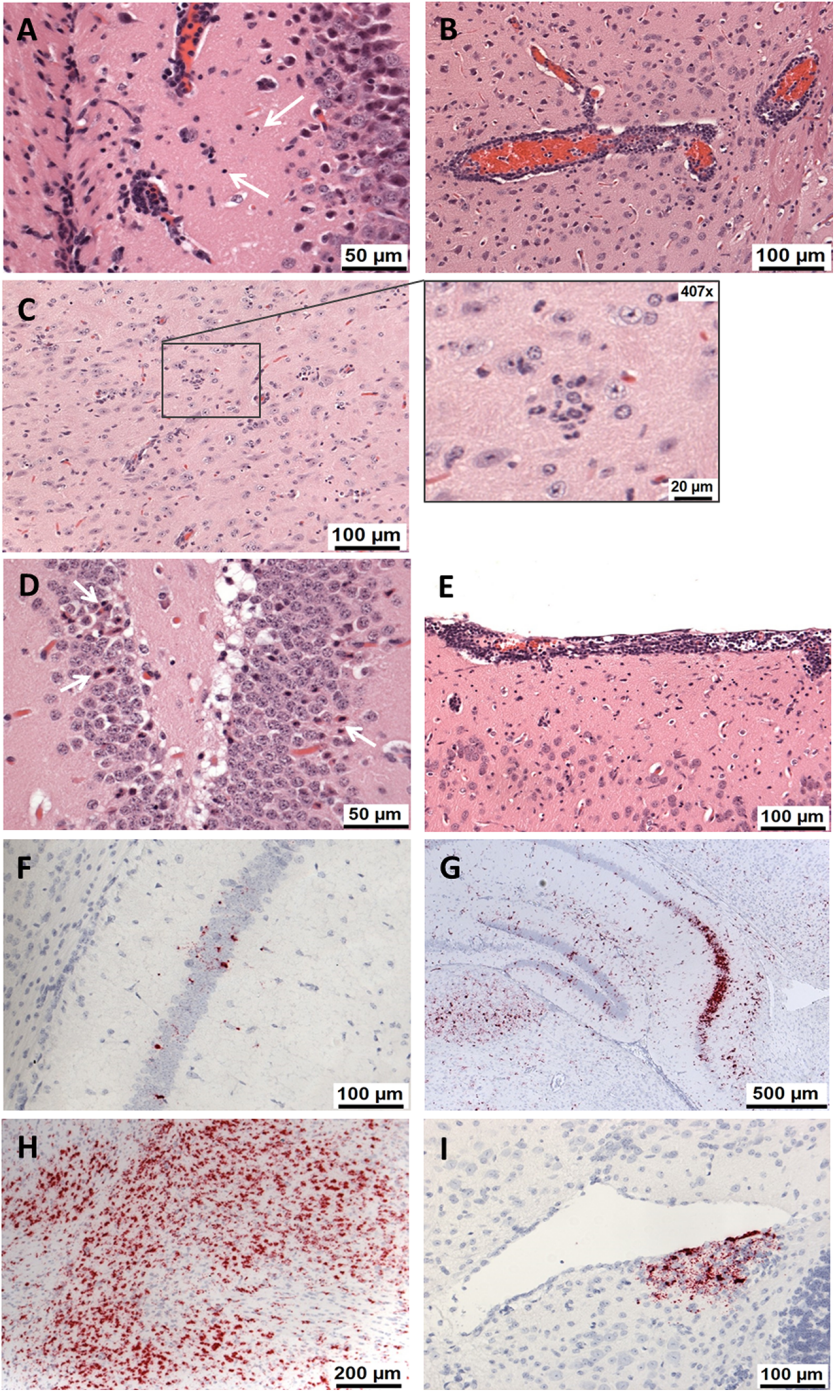
Histological and RNA *in situ* hybridisation findings in the brain of ZIKV-challenge A129 mice. (A) Scattered nuclear fragmentation in the hippocampus (Animal 86756, 10^6^ pfu ZIKV^AS^, day 7). (B) Perivascular cuffing by mononuclear cells (Animal 86762, 10^6^ pfu ZIKV^AS^, day 7). (C) Scattered polymorphonuclear cells (PMNs) in the neuropil, including higher magnification of PMNs (Animal 86722, 10^6^ pfu ZIKV^AF^, day 7). (D) Diffuse neuronal degeneration in Ammon’s horn of hippocampus (Animal 86724, 10^6^ ZIKV^AF^, day 6). (E) Infiltration of inflammatory cells, mainly mononuclear, in the meninges (Animal 86765, 10 pfu ZIKV^AF^, day 7). (F) Occasional scattered cells staining positive for viral RNA in the hippocampus (Animal 86780, 10^6^ pfu ZIKV^AF^, day 3). (G) Patchy to diffuse positive staining for viral RNA in the hippocampus (Animal 86783, 10^6^ pfu ZIKV^AF^, day 5). (H) Strong positive staining for viral RNA (Animal 86740, 10 pfu ZIKV^AF^, day 7). (I) Focus of positively staining cells for viral RNA in sub-ependymal area of the fourth ventricle (Animal 86773, 10^6^ ZIKV^AS^, day 5). A-E show sections stained with haematoxylin and eosin (H&E) and F-I show RNA *in situ* hybridisation images.

Brain lesions consistent with ZIKV infection were observed, variably, in animals from all challenged groups (Table 1). These comprised (i) nuclear fragmentation scattered diffusely within the grey and white matter (Fig 3A); (ii) perivascular inflammatory cell cuffing, mainly mononuclear cells (Fig 3B); (iii) widely distributed, scattered, occasional occurrence of polymorphonuclear leukocytes (PMNs) in the neuropil (Fig 3C) and perivascular location; (iv) the presence of scattered, partially degenerated cells in the neuron layer of the hippocampus (Ammon’s horn), comprising hyper-eosinophilic cytoplasms and irregularly shaped, partially condensed nuclei (Fig 3D); and (v) patchy meningeal infiltration by mainly mononuclear inflammatory cells (Fig 3E). Histological lesions were first observed in the ZIKV^AF^ groups on day 5 (high dose) and day 7 (low dose), ranging in severity from mild to moderate. By contrast, histological changes were not seen until 7 days post-challenge in the high dose ZIKV^AS^ infection group, and remained present at the day 14 endpoint of the study. Minimal changes only were seen at the day 14 time point in animals which received a low dose of ZIKV^AS^.

**Table 1:** Histological findings in brains of A129 mice infected with ZIKV.

Challenge (strain and dose)	Day post-challenge	Animal ID	Brain					Challenge (strain and dose)	Day post-challenge	Animal ID	Brain				
			Diffusely scattered nuclear debris	Lymphocytic perivascular cuffing	Diffusely scattered PMNs	Degenerating neurons–hippocampus	Patchy, meningeal infiltration by inflammatory cells				Diffusely scattered nuclear debris	Lymphocytic perivascular cuffing	Diffusely scattered PMNs	Degenerating neurons–hippocampus	Patchy, meningeal infiltration by inflammatory cells
ZIKV^AF^ (strain MP1751), 10 pfu	Day 1	86775	WNL	WNL	WNL	WNL	WNL	ZIKV^AS^ (strain PRVABC59), 10 pfu	Day 1	86737	WNL	WNL	WNL	WNL	WNL
		86774	WNL	WNL	WNL	WNL	WNL			86736	WNL	WNL	WNL	WNL	WNL
		86776	WNL	WNL	WNL	WNL	WNL			86735	WNL	WNL	WNL	WNL	WNL
	Day 3	86786	WNL	WNL	WNL	WNL	WNL		Day 3	86721	WNL	WNL	WNL	WNL	WNL
		86788	WNL	WNL	WNL	WNL	WNL			86718	WNL	WNL	WNL	WNL	WNL
		86741	WNL	WNL	WNL	WNL	WNL			86734	WNL	WNL	WNL	WNL	WNL
	Day 5	86772	WNL	WNL	WNL	WNL	WNL		Day 5	86732	WNL	WNL	WNL	WNL	WNL
		86771	WNL	WNL	WNL	WNL	WNL			86717	WNL	WNL	WNL	WNL	WNL
		86773	WNL	WNL	WNL	WNL	WNL			86716	WNL	WNL	WNL	WNL	WNL
	Day 7	86765	Min	**Mild**	**Mild**	WNL	**Mild**		Day 7	86715	WNL	WNL	WNL	WNL	WNL
		86740	**Mild**	**Mild**	**Mild**	Min	**Mod**			86730	WNL	WNL	WNL	WNL	WNL
		86784	Min	**Mild**	**Mild**	WNL	**Mild**			86729	WNL	WNL	WNL	WNL	WNL
	Day 14	Animals met humane endpoints prior to reaching this timepoint							Day 14	86714	WNL	Min	WNL	WNL	Min
										86728	WNL	WNL	WNL	WNL	WNL
										86727	WNL	WNL	WNL	WNL	WNL
ZIKV^AF^ (strain MP1751), 10^6^ pfu	Day 1	86764	WNL	WNL	WNL	WNL	WNL	ZIKV^AS^ (strain PRVABC59), 10^6^ pfu	Day 1	86719	WNL	WNL	WNL	WNL	WNL
		86766	WNL	WNL	WNL	WNL	WNL			86750	WNL	WNL	WNL	WNL	WNL
		86768	WNL	WNL	WNL	WNL	WNL			86751	WNL	WNL	WNL	WNL	WNL
	Day 3	86778	WNL	WNL	WNL	WNL	WNL		Day 3	86749	WNL	WNL	WNL	WNL	WNL
		86779	WNL	WNL	WNL	WNL	WNL			86748	WNL	WNL	WNL	WNL	WNL
		86780	WNL	WNL	WNL	WNL	WNL			86760	WNL	WNL	WNL	WNL	WNL
	Day 5	86777	**Mild**	**Mild**	**Mild**	WNL	Min		Day 5	86763	WNL	WNL	WNL	WNL	WNL
		86783	**Mild**	**Mild**	**Mild**	Min	**Mild**			86761	WNL	Min	Min	WNL	WNL
		86767	Min	**Mild**	**Mild**	Min	**Mild**			86745	WNL	WNL	WNL	WNL	WNL
	Day 7	Animals met humane endpoints prior to reaching this timepoint							Day 7	86762	Min	**Mod**	Min	WNL	Min
										86756	**Mod**	**Mod**	Min	Min	**Mod**
										86759	Min	**Mod**	Min	WNL	**Mild**
	Day 14	Animals met humane endpoints prior to reaching this timepoint							Day 14	86757	Min	**Mod**	WNL	WNL	**Mild**
										86758	Min	**Mild**	WNL	Min	Min
										86744	WNL	Min	Min	WNL	WNL

WNL, within normal limits; Min, minimal; Mod, moderate

In addition, samples were stained for the presence of ZIKV RNA within the brain tissue (Table 2). Viral RNA was initially detected at day 3 post-challenge in animals infected with both ZIKV strains (Fig 3F). In the ZIKV^AF^ groups, viral RNA staining was more prominent (Fig 3G and 3H) with time post-challenge; however, in the ZIKV^AS^-challenged animals, low levels of staining were only observed in some animals (Fig 3I).

**Table 2:** Viral RNA staining in tissues from A129 mice challenged with ZIKV.

Challenge (strain and dose)	Day post-challenge	Animal ID	Brain	Spleen	Liver	Testis	Heart	Lung	Kidney	Challenge (strain and dose)	Day post-challenge	Animal ID	Brain	Spleen	Liver	Testis	Heart	Lung	Kidney
ZIKV^AF^ (strain MP1751) 10 pfu	Day 1	86775	-	-	-	-	-	-	-	ZIKV^AS^ (strain PRVABC59) 10 pfu	Day 1	86737	-	-	-	-	-	-	-
		86774	-	-	-	-	-	-	-			86736	-	-	-	-	-	-	-
		86776	-	-	-	-	-	-	-			86735	-	-	-	-	-	-	-
	Day 3	86786	-	++	-	-	-	-	-		Day 3	86721	+	+	-	-	-	-	-
		86788	-	+	-	-	-	-	-			86718	+	+	-	-	-	-	-
		86741	-	+	-	-	-	-	-			86734	+	+	-	-	-	-	-
	Day 5	86772	+	+	+	+	++	+	++		Day 5	86732	+	++++	+	+	+	+	+
		86771	+	+++	+	+	++	++	++			86717	+	++++	+	+	+	-	+
		86773	+	++	+	++	++	++	++			86716	-	++++	+	-	+	-	-
	Day 7	86765	++++	++	+	++	+++	++	+		Day 7	86715	+	++	+	+	++	+	+
		86740	+++	++	+	++	++	+	+			86730	-	+	-	+	ND	-	+
		86784	++++	+++	++	++	+	++	+			86729	-	+	+	+	++	+	++
	Day 14	Animals met humane endpoints prior to reaching this timepoint									Day 14	86714	+	-	-	-	-	ND	-
												86728	-	-	+	-	-	-	-
												86727	-	-	-	-	-	-	-
ZIKV^AF^ (strain MP1751) 10^6^ pfu	Day 1	86764	-	+	-	-	-	-	-	ZIKV^AS^ (strain PRVABC59) 10^6^ pfu	Day 1	86719	-	+	-	-	-	-	-
		86766	-	+	-	-	-	-	-			86750	-	+	-	-	-	-	-
		86768	-	+	-	-	-	-	-			86751	+	+	-	-	-	-	-
	Day 3	86778	+	+++	-	-	-	-	-		Day 3	86749	-	++++	-	-	-	-	-
		86779	-	++++	-	-	-	-	-			86748	-	++++	-	-	-	-	-
		86780	+	++	-	-	-	-	-			86760	-	++++	-	-	-	-	-
	Day 5	86777	+++	++	+	ND	+++	++	++		Day 5	86763	+	+++	+	+	+	+	++
		86783	+++	++	+	++	++	++	+			86761	+	++	+	++	+	+	++
		86767	+++	+++	+	ND	+++	++	++			86745	-	+	+	++	+	+	+
	Day 7	Animals met humane endpoints prior to reaching this timepoint									Day 7	86762	+	+	-	+	+	+	+
												86756	+	+	-	++	+	+	-
												86759	+	+	-	++	+	-	+
	Day 14	Animals met humane endpoints prior to reaching this timepoint									Day 14	86757	+	+	-	++++	+	-	-
												86758	+	+	-	++++	-	-	-
												86744	+	+	-	++++	-	-	-

-, no staining; +, denotes intensity of staining; ND, not done (samples not collected)

### ZIKV challenge of A129 mice caused histological changes, associated with the infection, in the spleen, testis and the heart

In addition to changes in the brain, histological changes were also assessed in the spleen, testis, heart, liver, lung and kidney (Tables 2 and 3).

**Table 3:** Histological findings in spleen, liver, testis and heart of A129 mice infected with ZIKV.

Challenge (strain and dose)	Day post-challenge	Animal ID	Spleen			Liver	Testis	Heart	Challenge (strain and dose)	Day post-challenge	Animal ID	Spleen			Liver	Testis	Heart
			Poorly defined areas of white pulp with large MN cells	EMH +/- apoptosis	Mature PMNs in red pulp sinuses							Poorly defined areas of white pulp with large MN cells	EMH +/- apoptosis	Mature PMNs in red pulp sinuses			
ZIKV^AF^ (strain MP1751), 10 pfu	Day 1	86775	WNL	**Mild**	Min	WNL	WNL	WNL	ZIKV^AS^ (strain PRVABC59), 10 pfu	Day 1	86737	WNL	**Marked**	Min	Min	WNL	WNL
		86774	WNL	**Mod**	WNL	WNL	WNL	WNL			86736	WNL	**Mod**	Min	WNL	WNL	WNL
		86776	WNL	**Mod**	Min	WNL	WNL	WNL			86735	WNL	Min	Min	WNL	WNL	WNL
	Day 3	86786	WNL	**Mod**	**Mild**	WNL	WNL	WNL		Day 3	86721	WNL	**Mild**	WNL	WNL	WNL	WNL
		86788	WNL	WNL	**Mild**	Min	WNL	WNL			86718	WNL	Min	WNL	WNL	WNL	WNL
		86741	WNL	WNL	Min	WNL	WNL	WNL			86734	WNL	**Mod**	Min	WNL	WNL	WNL
	Day 5	86772	WNL	**Mild**	**Mod**	**Mild**	**Mild**	**Mod**		Day 5	86732	**Mild**	**Mod**	**Marked**	WNL	WNL	WNL
		86771	Min	**Marked**	**Mod**	WNL	Min	Min			86717	**Mild**	**Mod**	**Mod**	WNL	WNL	WNL
		86773	**Mild**	**Marked**	**Mod**	WNL	**Mild**	**Mild**			86716	**Mild**	**Marked**	**Mod**	WNL	WNL	Min
	Day 7	86765	**Mod**	**Mild**	**Marked**	Min	Min	**Mod**		Day 7	86715	Min	**Mild**	**Mild**	WNL	WNL	WNL
		86740	**Mild**	**Mod**	**Marked**	Min	Min	WNL			86730	Min	**Mild**	Min	WNL	WNL	ND
		86784	Min	**Mod**	**Mod**	Min	Min	Min			86729	**Mild**	**Mod**	**Mod**	WNL	WNL	Min
	Day 14	Animals met humane endpoints prior to reaching this timepoint								Day 14	86714	WNL	**Mild**	**Mild**	WNL	WNL	WNL
											86728	WNL	Min	Min	WNL	WNL	WNL
											86727	WNL	**Mod**	Min	WNL	WNL	WNL
ZIKV^AF^ (strain MP1751), 10^6^ pfu	Day 1	86764	WNL	**Mild**	Min	WNL	WNL	WNL	ZIKV^AS^ (strain PRVABC59), 10^6^ pfu	Day 1	86719	WNL	**Mod**	Min	WNL	WNL	WNL
		86766	WNL	**Mild**	Min	WNL	WNL	WNL			86750	WNL	Min	Min	WNL	WNL	WNL
		86768	WNL	**Mod**	Min	WNL	WNL	WNL			86751	WNL	**Mod**	**Mild**	Min	WNL	ND
	Day 3	86778	Min	**Mild**	**Mod**	WNL	WNL	Min		Day 3	86749	**Mild**	**Mod**	**Mod**	WNL	WNL	WNL
		86779	WNL	**Mod**	**Marked**	WNL	**Mild**	**Mild**			86748	**Mild**	**Marked**	**Marked**	WNL	WNL	WNL
		86780	Min	**Mod**	**Mod**	WNL	**Mild**	**Mild**			86760	WNL	**Mod**	**Mild**	WNL	WNL	WNL
	Day 5	86777	**Mod**	**Mod**	**Mod**	Min	ND	Min		Day 5	86763	Min	**Mod**	**Mod**	WNL	WNL	Min
		86783	**Mod**	**Marked**	**Mod**	Min	**Mild**	Min			86761	WNL	**Marked**	**Mild**	WNL	WNL	WNL
		86767	**Mild**	**Marked**	**Marked**	Min	ND	Min			86745	**Mod**	**Mod**	**Mild**	Min	WNL	WNL
	Day 7	Animals met humane endpoints prior to reaching this timepoint								Day 7	86762	WNL	**Mod**	Min	Min	Min	WNL
											86756	Min	**Marked**	Min	**Mod**	WNL	WNL
											86759	Min	**Mod**	Min	Min	WNL	WNL
	Day 14	Animals met humane endpoints prior to reaching this timepoint								Day 14	86757	Min	**Mod**	Min	Min	WNL	WNL
											86758	Min	**Mild**	Min	Min	WNL	WNL
											86744	WNL	**Mild**	**Mild**	Min	**Marked**	WNL

WNL, within normal limits; Min, minimal; Mod, moderate; EMH, extra-medullary haematopoiesis; PMN, polymorphonuclear cells; MN, mononuclear cells; MKC, megakaryocytes; ND, not done

**Fig 4:**
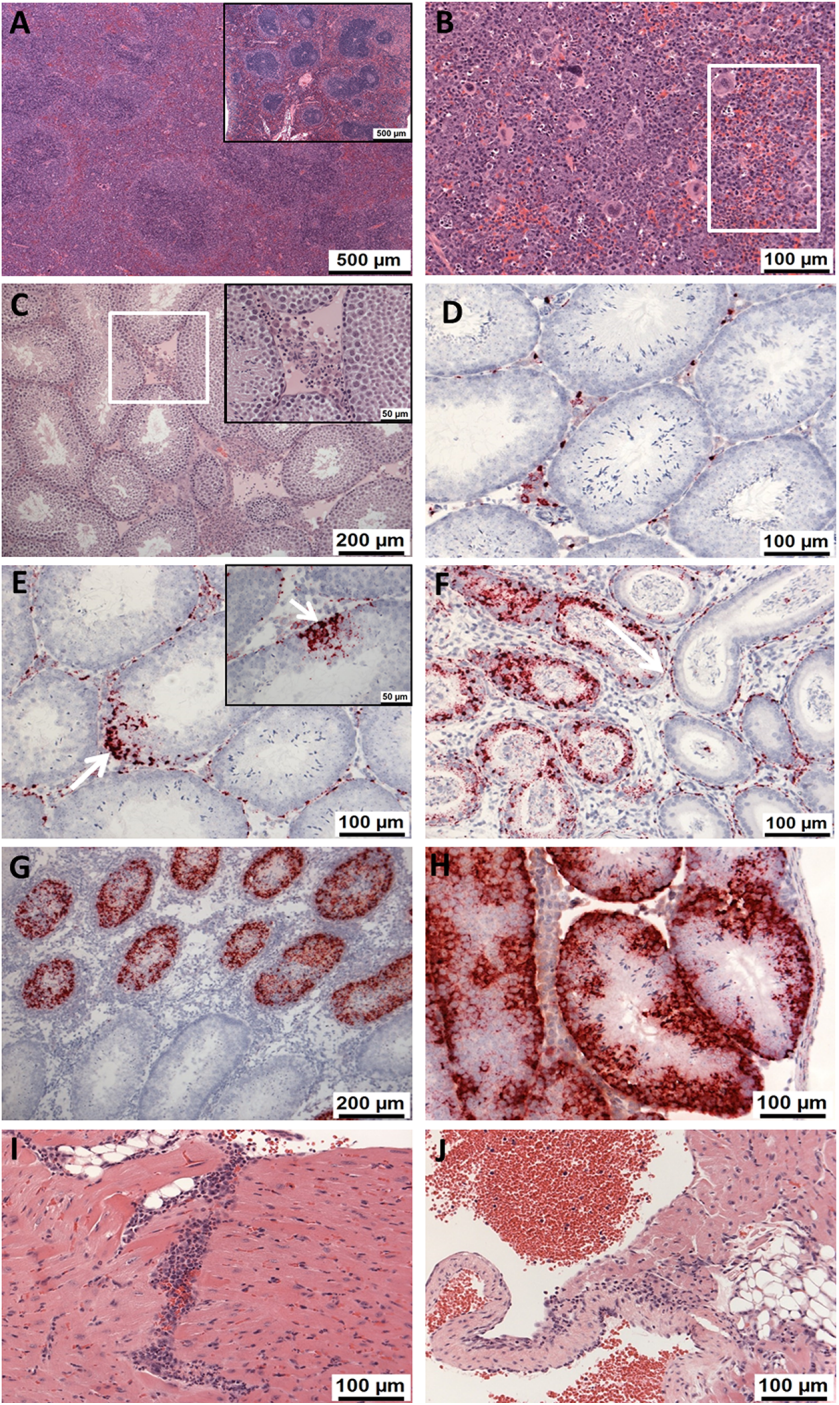
Histological and RNA *in situ* hybridisation findings in the spleen, testes and heart of ZIKV-challenge A129 mice. (A) Spleen. Poorly defined areas comprising large mononuclear cells within the white pulp (Animal 86783, 10^6^ pfu ZIKV^AF^, day 5). Inset, normal spleen with well defined, small germinal centres within the white pulp (Animal 86739, unchallenged). (B) Spleen. Prominent, extra-medullary haematopoiesis in the red pulp. Rectangle, numerous PMNs in the red pulp sinuses (Animal 86724, 10^6^ pfu ZIKV^AF^, day 7). (C) Testis. Expansion of the interstitial tissue by proteinaceous fluid, macrophages and PMNs. Inset, higher power image of area within white square (Animal 86772, 10 pfu ZIKV^AF^, day 5). (D) Testis. Mild infiltration of PMNs into the interstitial space with positive viral staining (Animal 86779, 10^6^ ZIKV^AF^, day 3). (E) Testis. Positive staining of virally infected cells focally within the walls of the seminiferous tubules (white arrows) as well as within the interstitium (Animal 86784, 10 pfu ZIKV^AF^, day 7). (F) Testis. Epididymis with positive staining of cells in lumen and epithelium of the efferent ductules as well as the interstitium (Animal 86784, 10 pfu ZIKV^AF^, day 7). (G) Testis. Positive staining of cells in the necrotic seminiferous tubules (Animal 86744, 10^6^ pfu ZIKV^AS^, day 14). (H) Testis. Intra-tubular and interstitial cell staining (Animal 86757, 10^6^ pfu ZIKV^AS^, day 7). (I) Heart. Infiltration of myocardium by macrophages and PMNs (Animal 86779, 10^6^ ZIKV^AF^, day 3). (J) Heart. Infiltration of an atrio-ventricular valve by macrophages and PMNs (Animal 86780, 10^6^ pfu ZIKV^AF^, day 3). A-C and I-J show sections stained with haematoxylin and eosin (H&E) and D-H show RNA *in situ* hybridisation images.

In the spleen, histological changes comprised (i) poorly defined areas comprising large mononuclear cells within the white pulp, with numerous apoptotic bodies and scattered mitotic figures (Fig 4A); (ii) prominent, extra-medullary haematopoiesis (EMH) in the red pulp with numerous precursor cells, apoptotic bodies and scattered megakaryocytes (Fig 4B); and (iii) numerous, mature PMNs within the red pulp sinuses (Fig 4B). The changes observed in all animals sampled at day 1 post-challenge consisted of increased EMH, considered to be a non-specific response to the virus. Histological changes more likely related to the viral infection, namely the poorly defined area comprising large mononuclear cells within the white pulp, were first detected at day 3. By day 14 post-challenge, reduced severity of changes and viral RNA staining was observed in ZIKV^AS^ infected animals compared to the previous time points suggesting recovery in this organ.

In the testis, in a proportion of ZIKV-challenged animals, the interstitial tissue was infiltrated by macrophages and sometimes PMNs. Homogeneous, eosinophilic material, interpreted as proteinaceous fluid was also observed expanding the interstitium variably (Fig 4C). In some animals, necrosis of the seminiferous tubules was noted. After challenge with ZIKV^AF^, changes in the testis were first recorded on day 3, concomitant with the detection of viral RNA. Virus was evident in the interstitial tissues (Fig 4D). By day 7, viral RNA was observed multifocally within the seminiferous tubules (Fig 4E). In one animal euthanised at day 7, epididymis was present, with prominent viral staining observed in the interstitium of the testis and epididymis, and focally in the tubular epithelium and lumena of the efferent tubules (Fig 4F). In the groups infected with ZIKV^AS^, histological changes were noted in only one animal culled on day 14. However, viral RNA was detected from day 5 in both low and high dose challenge groups. In the low dose group viral RNA was not detected at day 14, but in those challenged with the high dose, viral RNA staining had increased substantially to day 14. The virus was present in necrotic seminiferous tubules (Fig 4G) and intra-tubular cells as well as the interstitium (Fig 4H). Therefore, following both ZIKV^AF^ and ZIKV^AS^ infection, there was clear evidence that the virus crossed the blood/testis barrier.

In the heart, histological changes were observed in several animals challenged with ZIKV^AF^, but minimal effects were only observed after infection with ZIKV^AS^. These comprised macrophages and PMNs infiltrating the myocardium (Fig 4I), occasionally associated with cardiomyocyte degeneration and/or nuclear debris. In addition, infiltration of the atrio-ventricular valves and connective tissue surrounding the epicardium, by similar inflammatory cells was observed (Fig 4J). Viral staining was noted after challenge with both ZIKV strains from day 7, but by day 14, staining was present only in one of the animals that had been challenged with a high dose ZIKV^AS^.

Changes considered to be directly attributable to ZIKV infection were not detected in the liver and lung; nevertheless viral RNA was detected in these organs. In the kidney, where histological changes were not detected, ZIKV RNA was found within the cortical and medullary interstitium.

### Using a recent isolate, ZIKV^AS^ remained non-lethal in A129 mice and showed similar responses to the previously used contemporary strain

The observation that a contemporary strain of ZIKV^AS^ (PRVABC59) did not cause clinical disease in A129 mice, led us to test another strain from the same lineage. For this work, we used a strain (ZIKV^AS^-PHE) recently isolated from a returning UK traveller who had visited Guadeloupe [38].

**Fig 5:**
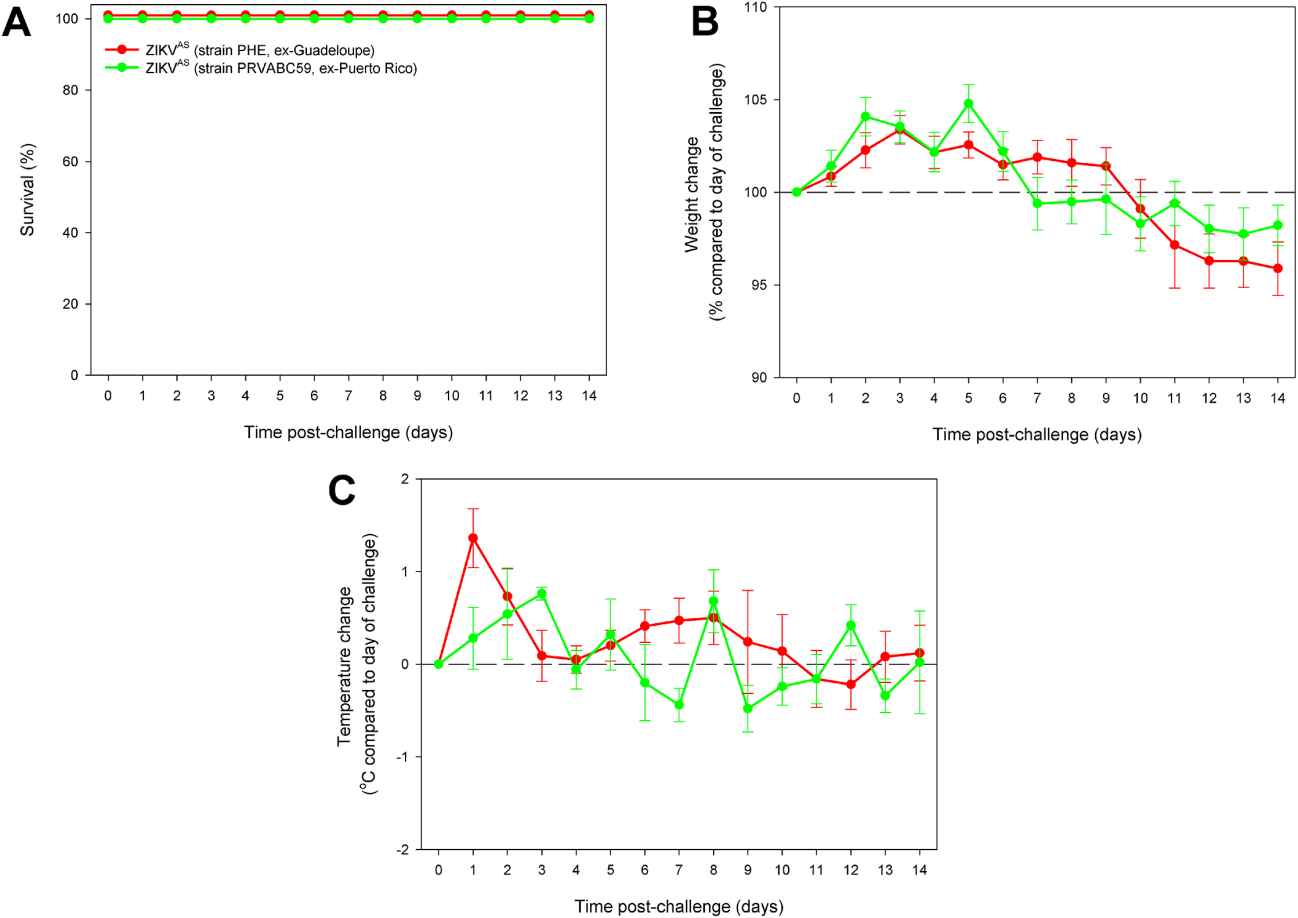
Clinical data from A129 mice challenged with different strains of ZIKV^AS^. 5–8 week old A129 mice were subcutaneously challenged with 10^6^ pfu ZIKV^AS^ virus. (A) Kaplin-Meier survival plot. (B) Differences in weight compared to date of challenge. (C) Temperature change compared to date of challenge. Graphs B and C show the mean values with error bars denoting standard error. Group sizes were n = 5.

**Fig 6:**
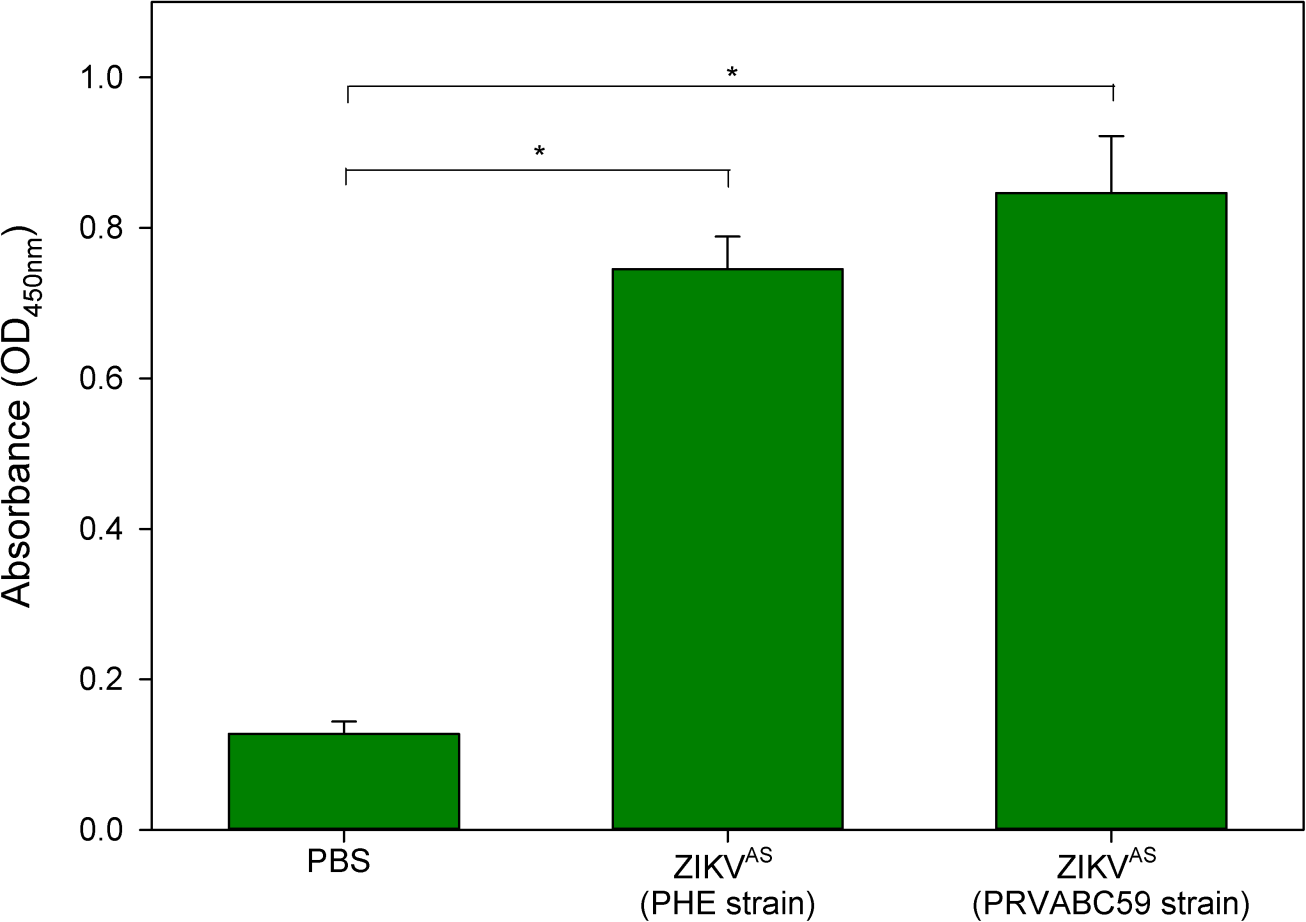
Seroreactivity data of A129 mice challenged with different strains of ZIKV^AS^. Sera collected 14 days post-challenge were assessed for antibody responses to ZIKV. * indicates statistical significance (P<0.05, Mann-Whitney test).

Results from challenged A129 mice confirmed the previous finding with ZIKV^AS^; neither isolate caused lethality (Fig 5A). Weight differences and temperatures were also similar between animals treated with the two ZIKV^AS^ isolates (Fig 5B and 5C, respectively), although with both strains there was a rapid weight loss of ≈5% over 2–3 days before weight stabilisation. Clinical signs were not observed in either of the challenged groups. At the end of the study, sera from culled animals were assessed for antibody levels to confirm seroreactivity. All of the ZIKV^AS^-challenged animals had detectable antibody responses (Fig 6).

Histological lesions and *in situ* detection of viral RNA was conducted in the brain, testis and heart (Table 4). Microscopic changes referable to infection by ZIKV were observed in the brain and testis of a proportion of animals in both groups. Only minimal microscopic changes were observed in the heart of a single animal. Viral RNA was also detected in the brain and testis of a proportion of animals from both groups. In the brain, changes were mainly minimal with scant staining of cells in two animals from each group. Strong viral RNA staining was noted in the testis of animals in both groups. Generally, staining patterns comprised mild staining of interstitial cells or/and strong staining of cells within the seminiferous tubules, the latter supportive of virus crossing the blood:testis barrier. In the heart, viral RNA was detected only in samples collected on day 7 post-challenge. There did not appear to be prominent difference in the prevalence and severity of changes in animals between the groups infected with the different ZIKV^AS^ strains.

**Table 4:** Histological and *in situ* detection of viral RNA in A129 mice challenged with two different strains of ZIKV^AS^.

Challenge strain and time of sample	Animal ID	Brain				Testis			Heart	
		Diffusely scattered nuclear debris	Lymphocytic perivascular cuffing	Patchy, meningeal infiltration by inflammatory cells	Level of viral RNA staining	M’phages and/or PMNs +/- oedema in interstitium	Inflammation with tubular degeneration and necrosis	Level of viral RNA staining	PMNs in myocardium +/- scattered nuclear debris	Level of viral RNA staining
ZIKV^AS^ (strain PHE), Day 7	86612	WNL	WNL	WNL	-	WNL	WNL	**++** i.s.	WNL	**+**
	86635	WNL	WNL	WNL	-	WNL	WNL	**++** i.s.	WNL	**+**
	86634	WNL	WNL	WNL	-	WNL	WNL	**++** i.s.	WNL	**+**
	86637	WNL	WNL	WNL	-	WNL	WNL	**++** i.s.	WNL	**+**
	86613	WNL	WNL	WNL	-	WNL	WNL	**++** i.s.	WNL	**++**
ZIKV^AS^ (strain PHE), Day 14	86621	Min	Min	Min	-	WNL	WNL	-	WNL	-
	86623	**Mild**	**Mild**	WNL	**+**	**Mod**	WNL	**++++** i.t.	WNL	-
	86619	WNL	WNL	WNL	-	WNL	WNL	**+** i.s. and i.t.	WNL	-
	86624	WNL	WNL	WNL	-	WNL	WNL	-	WNL	-
	86622	**Mild**	Mod	**Mod**	**+**	**Mild**	**Mild**	**++++** i.t.	WNL	-
ZIKV^AS^ (strain PRVABC59), Day 14	86625	Min	Min	Min	**+**	WNL	**Marked**	**++++** i.t.	Min	-
	86614	Min	**Mild**	WNL	-	WNL	**Mod**	**++++** i.t.	WNL	-
	86615	Min	**Mild**	Min	-	WNL	**Mod**	**++++** i.t.	WNL	-
	86617	WNL	WNL	WNL	-	WNL	WNL	-	WNL	-
	86620	WNL	Min	WNL	**+**	WNL	WNL	-	WNL	-

WNL, within normal limits; Min, minimal; Mod, moderate; +, denotes intensity of viral RNA staining; PMN, polymorphonuclear cells; i.s., interstitial; i.t., intra-tubular

## Discussion

In the present study and A129 mouse model was used to compare the virulence of 2 lineages of ZIKV; African (ZIKV^AF^) and Asian (ZIKV^AS^). Infection with ZIKV^AF^ was lethal in A129 mice whereas infection with ZIKV^AS^ was well tolerated. For both lineages, viral RNA and pathological changes were detected mainly within the brain, spleen and testis. Using a similar mouse model, but from a different parental background (*Ifnar1*^*-/-*)^, ZIKV-challenged animals sustained high viral loads in the brain and testes [30]. However, unlike in the A129 model, after infection with 100 focus-forming units (FFU) of ZIKV^AS^ via the subcutaneous route, all *Ifnar1*^*-/-*^ animals perished within 10 days [30]. The lethality of ZIKV in this mouse model was further confirmed using different strains of ZIKV^AF^ and ZIKV^AS^ [30]. This difference might be attributable to the parental mouse strains used to generate *Ifnar1*^*-/-*^ mice, since it is known for example that susceptibilities to viruses between laboratory strains vary [39]. A further related complication of using *Ifnar1*^*-/-*^ mice is their genetic background. Whilst initial studies of the *Ifnar1-/-* model were set up in Balb/c mice [40], work with ZIKV has been undertaken in mice with C57BL/6 backgrounds [30, 31]. The parental background of *Ifnar1-/-* may subsequently affect results, particularly as C57BL/6 and Balb/c are prototypical Th1- and Th2-type mouse strains, respectively [41]. The challenge route of infection is also important, as the intraperitoneal route results in a different outcome to when virus is delivered subcutaneously [42]; the latter being the preferable route to resemble the natural route of transmission via mosquito bite.

Although differences in lethality were observed between the present studies and those in *Ifnar*^*-/-*^ mice [30], the present studies confirmed the wide distribution of viral RNA in the tissues of ZIKV^AS^ challenged mice. The finding of pathological changes in the brain is consistent with other reports, including those dating back to the 1970s [43]. The finding of neurotropism of the virus should enable research on brain effects to be undertaken in follow-up studies using subcutaneous inoculation instead of relying on direct, intracranial inoculations as used by others [44]. Evidence of ZIKV infection in the testis of mice, after challenge, has also been reported by others [28–30]. The data in A129 mice indicate damage to the seminiferous tubules, infiltration of inflammatory cells in the interstitium and breakdown of the blood:testis barrier as observed in *Ifnar1*^*-/-*^ mice [29] and other similar mouse models where virus has been detected in seminal fluid [45]. In the interstitium, the observations support the finding that virus is present in semen after human ZIKV infection [46]. Mice with defective IFN signalling have also been shown to be highly susceptible to infection via the vaginal route [47]. Therefore, the A129 mouse might be considered for modelling the sexual transmission route of ZIKV, in addition to looking at mosquito-borne infection routes.

Whilst A129 mice do have some form of immunological deficit, they are not as immunocompromised as AG129 mice which have also been shown to be highly susceptible to ZIKV infection [48]. In the AG129 model, tissue damage to the brain was observed but there was no obvious damage to other organs examined (including the heart, liver, spleen, kidney and lung) [48]. In contrast, in the present studies, A129 mice additionally demonstrated extensive damage to the spleen and changes in the heart. For testing of vaccines, the A129 model has value because it retains the type-II interferon (IFN-γ) response, and it has been used to demonstrate protective vaccine efficacy with other arboviruses [49–51]. Additionally, unlike *Ifnar1*^*-/-*^ mice which are not widely obtainable and require breeding in specialised animal care facilities, A129 mice are commercially available with consistent standard genetic backgrounds.

The use of different lineages of ZIKV will be important in the assessment of pathogenicities of disease and efficacies of interventions. ZIKV^AF^ was widely available at the beginning of the recent outbreak, and was widely used for initial studies [27, 44]. However, during the WHO-declared period of ZIKV being a Public Health Emergency of International Concern (PHEIC), ZIKV^AS^ strains were also made widely available. The strains of ZIKV^AS^ used for our studies included PRVABC59 (GenBank Accession number KU501215), a virus derived from the US Centres for Disease Control [52] and widely distributed to other laboratories, including as part of the Zika response by the Global Health Security Action Group (GHSAG). The strain has been used for demonstrating vaccine efficacy in mice [53] and NHPs [54]. PRVABC59 has also been used in NHP studies demonstrating the secretion of ZIKV in saliva [55]. Given that PRVABC59 has been used across mouse and NHP models, it is a strong candidate for use as the prototype ZIKV^AS^ strain to ensure consistency across studies and eliminate variation between strains. The concordance of results between the isolated PRVABC59 strain and one recently isolated from a patient [38] increases confidence that the A129 model is not lethal after ZIKV^AS^ challenge. Studies in NHPs have also demonstrated similar findings between the PRVABC59 strain [55] and virus stocks isolated from the same lineage [56, 57]. Given that the percent nucleotide identity among all the Western hemisphere ZIKV strains is >99% [52], the findings of similar pathogenicity to two ZIKV^AS^ strains in A129 mice is not surprising.

The stark difference in lethality and severity of disease between ZIKA^AF^ and ZIKV^AS^ infections warrants further investigation, including the effects of virus passage history on pathogenicity. However, the due to historic ZIKV^AF^ strains being propagated in newborn mice the alternative approach of isolating ZIKV^AS^ in newborn mice would be required to ascertain whether early events during virus isolation affect the virus characteristics. Indeed, the implications to human infection could be valuable and help with identifying future traits that may occur if the virus is skewed towards a particular lineage. Given that these viruses are approximately 88.8% identical / 97% amino acid (Table 5), further insights into the molecular determinants of disease should be investigated. This should be aided by recent development in reverse genetics platforms for ZIKV [58, 59].

**Table 5:** Genetic sequence similarities between ZIKV strains using the study.

	Percentage of polyprotein nucleic acid sequence (amino acid sequence)		
	ZIKV^AF^ MP1751	ZIKV^AS^ PHE	ZIKV^AS^ PRVABC59
ZIKV^AF^ MP1751	***	88.9 (97.1)	88.8 (97.1)
ZIKV^AS^ PHE	12.3 (2.9)	***	99.5 (99.8)
ZIKV^AS^ PRVABC59	12.5 (2.9)	0.5 (0.2)	***

Percent similarity is shown in upper right section, percent divergence in lower left.

## Materials and methods

### Ethics statement

All procedures with animals were undertaken according to the United Kingdom Animals (Scientific Procedures) Act 1986. These studies were approved by the ethical review process of Public Health England, Porton Down, UK, and by the Home Office, UK via an Establishment Licence (PEL PCD 70/1707) and project licence (30/3147). A set of humane end points based on clinical manifestation of disease were defined in the protocol of the project licence and are described below.

### Cells

Vero cells (African green monkey kidney epithelial cells) (European Collection of Cell Cultures, UK) were maintained in Dulbecco’s Modified Eagle Medium containing GlutaMAX (Invitrogen) and supplemented with 2% heat-inactivated foetal bovine serum (Sigma) at 37°C with 5% CO_2_.

### Viruses

ZIKV^AF^ strain MP1751 (Uganda, 1962) isolated by up to 3 passages in newborn mouse brain from pools of *Aedes africanus* mosquitoes [2] was obtained from the National Collection of Pathogenic Viruses (NCPV), UK. The passage history prior to deposit with NCPV included up to four passages between 1962–1972, by an unknown method. This was followed by one passage in Vero cells in 2011. ZIKV^AS^ strain PRVABC59 (Puerto Rico, 2016) was obtained from the US Centres for Disease Control, and had been passaged 4 times in Vero cells. ZIKV^AS^-PHE was isolated at Public Health England [38] in C6/36 cells (an *Aedes Albopictus*-derived cell line) and made available via NCPV and European Virus Archive goes Global (EVAg) collections. ZIKV stocks were propagated in Vero cells after inoculating at a multiplicity of infection (pfu/ml) of 0.01 and harvesting supernatant after 72 hr. Virus stocks were titrated by plaque assay on Vero cells. Foci of plaques were detected at 72 hr, following fixation with 10% formalin solution and staining with 2% crystal violet.

### Mouse experiments

Male mice (aged 6–8 weeks) with deficiencies in their type-I IFN receptor [60] were purchased from B&K Universal (A129). Mice were subcutaneously inoculated with 40 μl of virus suspension into each of the hind legs towards the ankle. Virus contained in the 80 μl inoculum volume equated to 10, 10^2^, 10^3^, 10^4^, 10^5^ or 10^6^ pfu for the dose reduction study, and 10 or 10^6^ pfu for the pathogenicity studies. Virus suspension was back-titrated in Vero cells to confirm dose concentration. Survival, temperature, weights and clinical signs were monitored for up to 14 days post-challenge. For clinical signs numerical scores were assigned (0, normal; 2, ruffled fur; 3, lethargy, pinched, hunched, wasp-waisted; 5, laboured breathing, rapid breathing, inactive, neurological; and 10, immobile). Temperatures were recorded by indwelling temperature chips. Animals reaching a clinical score >10 were terminated immediately and a weight loss of 20% or 10% in combination with any clinical sign was also used to indicate a humane end-point. At days 1, 3, 5 and 7 post-challenge, 3 mice from each group in the pathogenicity study were culled to assess local responses. All surviving animals were culled at day 14 post-challenge. Group sizes are stated in the relevant figure legends and the data representative of a single biological replicate.

### Measurement of viral burden

At necropsy, samples of spleen, liver, brain, kidney, lung, testis, heart and saliva were collected and immediately frozen at -80°C for virological analysis. Blood was collected into RNAprotect tubes (Qiagen) and rectal swabs were placed in 0.5 ml DMEM media (Sigma). Tissue samples were weighed and homogenised in phosphate buffered saline (PBS) using ceramic beads and an automated homogeniser (PreCellys). Tissue samples and biological fluids (blood, rectal swabs and saliva) were extracted using the RNeasy mini extraction kit (Qiagen). A ZIKV specific real-time RT-PCR assay was utilised for the detected of viral RNA using a published primer set [61]. Reactions were run and analysed on the 7500 Fast platform (Life Technologies). Quantification of viral load in samples was performed using a dilution series of quantified RNA oligonucleotide (Integrated DNA Technologies). Viral burden was expressed as genome copies per gram or per ml.

### Histological processing

Samples of brain, spleen, liver, heart, testis, kidney and lung were fixed in 10% neutral buffered saline and processed routinely to paraffin wax. Sections were cut at 3–5 μm, stained with haematoxylin and eosin (H&E) and examined microscopically. Lesions referable to infection were scored subjectively using the following scale: within normal limits, minimal, moderate and marked. The pathologist was blinded to the groups in order to prevent bias.

### RNA *in situ* hybridisation (ISH)

RNA ISH was performed with an RNAscope 2.5 (Advanced Cell Diagnostics) according to the manufacturer’s instructions. In brief, formalin-fixed paraffin-embedded tissue sections were deparaffinised by incubation for 60 min at 60°C. Hydrogen peroxide treatment for 10 min at room temperature quenched endogenous peroxidases. Slides were then boiled for 15 min in RNAscope Target Retrieval Reagents and incubated for 30 min in RNAscope Protease Plus before hybridisation. For probes, V-ZIKA-pp-O1-sense (Advanced Cell Diagnostics, catalogue no. 463791) and V-ZIKA-pp-O2-sense (Advanced Cell Diagnostics, catalogue no. 464541) were used for studies with ZIKV^AF^ and ZIKV^AS^ with 99% and 100% specificities, respectively. Tissues were counterstained with Gill’s haematoxylin and visualised with standard bright-field microscopy. For the brain, between 4–5 sections were examined. For the remaining tissues, 1 section of each was examined. Each slide was scanned systematically so all areas of the tissue were assessed.

### Assessment of antibody responses

A commercial ELISA kit was used to assess antibody responses against ZIKV (EI 2668–960; EuroImmun, Germany). Manufacturers guidelines were followed with the exception that due to the kit being developed for human samples, the detector antibody was changed to a goat anti-mouse IgM+IgG+IgA (AP501A; Millipore, UK). Following completion of staining, absorbance reading were read at a wavelength of 450nm using a plate spectrophotometer.

### Statistical analysis

Differences in RNA levels between the groups were statistically compared using Minitab (version 16.2.2). Due to the small group sizes (n = 3/group) and data not being normally-distributed, the nonparametric Mann-Whitney statistical test was used. Statistical significance was where P = 0.0801 (the lowest P-value obtainable using the conditions of n = 3/group).
